# Supplementary material for: Nutritional Profiles of Non-Dairy Plant-Based Cheese Alternatives
Source: Nutrients. 2022 Mar 16;14(6):1247. doi: 10.3390/nu14061247 (PMC8952881; doi:10.3390/nu14061247)
Supplement: Supplementary file 1 [file nutrients-14-01247-s001.zip › nutrients-1625866-supplementary.pdf]

Table S1. 2020 Sales Data for 7 Leading Brands of Non-dairy Cheeses in USA *

Company	Sales in $millions	Varieties of cheese	# with Ca fortification	# with Vit D fortification	# with vit B12 fortification	# with 5 g or more of protein
Brand A	83.6	17	11	0	3	0
Brand B	43.8	19	7	0	0	0
Brand C	29.8	21	1	0	21	0
Brand D	24.5	8	0	0	0	0
Brand E	13.7	22	7	0	0	0
Brand F	8.8	3	3	0	0	0
Brand G	5	10	3	0	0	0
Totals for Brand A-G		100	32	0	24	0
% of 245 cheeses		41%	13%	0	10%	0

* ref. 20

Table S2. Mean (Quartile 1–Quartile 3) of 5 nutrients per serving for non-dairy and dairy cheeses in different formats *.

Cheese Format	n	Calories	Sat fat	Sodium	Protein	Calcium
**Non–dairy**						
Blocks and Wheels	11	80 (75–125)	2 (1.5–4)	190 (150–215)	3 (1–4)	0 (0–1)
Slices	4	60 (60–62.5)	4 (3.875–4.125)	180 (177.5–202.5)	0 (0–0.75)	5 (0–10)
Shreds	5	80 (70–90)	3.5 (2.5–5)	250 (230–260)	1 (0–1)	0 (0–2)
Others	7	90 (70–95)	3.5 (1–6)	170 (142.5–210)	2 (0–3)	2 (1–4)
Total	27	80 (60–100)	3.5 (1.5–4.75)	190 (170–240)	1 (0–3)	0 (0–4)
**Dairy**						
Blocks and Wheels	11	70 (70–80)	3.5 (2.75–3.75)	190 (97.5–220)	7 (5.5–8)	15 (9–20)
Slices	14	70 (60–70)	2.5 (2.5–3)	122.5 (97.5–165)	6 (5.25–6.75)	15 (10–15)
Shreds	10	80 (70–80)	2.5 (1–3)	185 (170–225)	8 (7.25–8)	20 (15–20)
Ricotta & Farmers Cheese	6	80 (80–80)	2.5 (2.5–2.875)	60 (33.75–63.75)	5 (4–6)	10 (8.5–13.75)
Feta	5	50 (35–70)	2 (0–2.5)	340 (340–340)	6 (6–6)	8 (8–10)
Snacks	14	60 (50–70)	2.5 (2–3)	160 (140–160)	6 (5–6)	10 (10–10)
Spreadable & Brie	6	47.5 (37.5–57.5)	2.25 (1.25–2.875)	170 (152.5–180)	2 (2–2)	10 (5.5–10)
**Total**	66	70 (60–77.5)	2.5 (2–3)	160 (120–190)	6 (5–7)	10 (10–15)

* Statistical analyses were performed on the raw data presented in ref. 20.

Table S3. Percentage of dairy and non-dairy cheeses meeting or exceeding a nutrient guideline per serving *.

Nutrient Parameter	Dairy	Non-dairy
At least 5 g protein	83	11
At least 10% DV of Calcium	80	19
Over 1 g saturated fat	88	81
230 mg of sodium or more	15	33

* Statistical analyses were performed on the raw data presented in ref. 20
